# Physician Visiting List, Etc.

**Published:** 1852-07

**Authors:** 


### THE PHYSICIAN'S VISITING LIST, DAIRY AND BOOK OF ENGAGEMENTS.

The above title introduces to our notice a small but very useful book for the physician, as also the Dentist, under many circumstances, being as purported in the title, a diary of cases occurring of interest and engagements. We take the liberty of extracting the following article upon poisons and their proper antidotes, feeling assured it will richly repay the time spent in its perusal.

### POISONS.

In all cases of poisoning, the first step is to evacuate the stomach, which should be effected by one of those emetics which is most powerful and speedy in its operation, as sulphate of zinc, or sulphate of copper. When vomiting has already taken place, copious draughts of warm water or mucilaginous drinks should be given, to keep up the effect till the poisonous substance has been evacuated. If vomiting cannot be produced the stomach-pump must be used.

Inflammation of the stomach, congestion of the brain, and other symptoms are to be treated on general principles, viz: by blood-letting, cold applications, revulsives, cool mucilaginous drinks, milk, lime-water, &c. When prostration exists, stimulants should be resorted to, as in other cases.

The following is a list of the usual poisoning substances, with the appropriate remedies.

| POISONS. | ANTIDOTES. |
|---|---|
| *Acids* | *The Alkalies.* Common soap (soft or hard) in solution is an efficient remedy, and has the advantage of being always at hand. It should be followed by copious draughts of tepid water or flaxseed tea. For *nitric* and *oxalic* acids, the carbonates of magnesia and lime (chalk and water) are the best antidotes. When sulphuric acid has been taken, the use of much water will be improper. |
| *Alkalies and their salts.* | *The Vegetable Acids.* Common vinegar being always at hand, is most frequently used. The fixed oils, as castor, flaxseed, almond, and olive oils, form soaps with the alkalies, and thus also destroy their caustic effect. They should be given in *large quantity.* |
| EARTHS. *Baryta and its salts. Lime.* | *Epsom or Glauber's Salts,* in solution, or diluted sulphuric acid. The fixed oils also have the same effect as with the alkalies proper, when not in a compound state. |
| *Iodine. Iodide of Potassium.* | *Starch,* or wheat flour, in large quantities well mixed with water. For iodide of potassium there being no antidote, vomiting must be promoted by copious draughts of warm water. |

| POISONS. | ANTIDOTES. |
| --- | --- |
| *Antimony and its salts.* | *Astringent Infusions*, as of galls, oak bark, Peruvian Bark, or green tea, very strong. |
| *Arsenic and its compounds.* | *Hydrated Peroxide of Iron*, in tablespoonful doses every 5 or 10 minutes. |
| *Bismuth and its compounds.* | *Albumen.* Copious draughts of milk, combined with sweet mucilaginous drinks. |
| *Copper and its compounds.* | *Albumen*, as milk or whites of eggs in solution should be freely administered. *Vinegar* must be avoided. |
| *Gold, salts of.* | *Sulphate of Iron*, with a free use of mucilaginous drinks. |
| *Iron, salts of.* | *Carb. of Soda*, with mucilaginous drinks. |
| *Lead, salts of.* | *Sulphate of Magnesia*, (Epsom salts,) or diluted sulphuric acid. |
| *Mercury, salts of.* | *Albumen*, as whites of eggs, milk, or wheat flour beaten up with water. |
| *Silver, salts of.* | *Common Salt* (chloride of sodium) largely given. |
| *Tin, salts of.* | *Albumen.* Whites of eggs, milk, or flour. |
| *Zinc, salts of.* | *Albumen*, or carbonate of soda, with copious draughts of warm water, and especially milk. |
| *Phosphorus.* | *Magnesia* with water, and copious draughts of mucilaginous drinks. |
| *Gases.* | *Ammonia* cautiously inhaled is recommended for chlorine. Asphyxia produced by carbonic acid or carbonic oxide gases or sulphuretted hydrogen, must be treated by copious effusions of cold water especially to the head, blood-letting, artificial respiration, stimulants carefully administered, &c. |
| *Creasote.* | *Albumen* or whites of eggs, milk or wheat flour. |
| *Alcohol or spirituous* | A powerful emetic should be given, followed by copious draughts of warm water. |

| POISONS. | ANTIDOTES. |
| --- | --- |
| *liquors.* | Congestion of the brain, and other symptoms to be treated on general principles. |
| *Opium and other narcotics.* | The chief reliance to be placed on the most active emetics, (as tartar emetic, sulphate of copper, or sulphate of zinc,) and the stomach pump. Emetics are preferable to the stomach pump when the narcotic has been taken in substance. The patient should be kept in motion, and cold water dashed on the head and shoulders. Blood-letting may become necessary to relieve digestion. After other remedies fail, artificial respiration should be resorted to. |

